# A 12-Lead ECG database to identify origins of idiopathic ventricular arrhythmia containing 334 patients

**DOI:** 10.1038/s41597-020-0440-8

**Published:** 2020-03-23

**Authors:** Jianwei Zheng, Guohua Fu, Kyle Anderson, Huimin Chu, Cyril Rakovski

**Affiliations:** 1 Chapman University, Orange, USA; 2 Ningbo First Hospital of Zhejiang University, Ningbo, China

**Keywords:** Arrhythmias, Arrhythmias, Electrodiagnosis

## Abstract

Cardiac catheter ablation has shown the effectiveness of treating the idiopathic premature ventricular complex and ventricular tachycardia. As the most important prerequisite for successful therapy, criteria based on analysis of 12-lead ECGs are employed to reliably speculate the locations of idiopathic ventricular arrhythmia before a subsequent catheter ablation procedure. Among these possible locations, right ventricular outflow tract and left outflow tract are the major ones. We created a new 12-lead ECG database under the auspices of Chapman University and Ningbo First Hospital of Zhejiang University that aims to provide high quality data enabling detection of the distinctions between idiopathic ventricular arrhythmia from right ventricular outflow tract to left ventricular outflow tract. The dataset contains 334 subjects who successfully underwent a catheter ablation procedure that validated the accurate origins of idiopathic ventricular arrhythmia.

## Abstract (summary)

**Table:** 

Measurement(s)	Ventricular arrhythmia
Technology Type(s)	electrocardiography
Factor Type(s)	sex
Sample Characteristic - Organism	Homo sapiens

Machine-accessible metadata file describing the reported data: 10.6084/m9.figshare.11926506

## Background & Summary

Originating from the two lower chambers (the ventricles) of the heart, a premature ventricular complex (PVC) causes an extra, or abnormal, heartbeat that occurs earlier than it should. Ventricular tachycardia (VT) manifested by three or more consecutive PVCs are seen at a rate of 100 bpm or higher. For healthy people, an occasional period of PVCs is not a concern and typically does not require treatment. However, for those with underlying health conditions, PVC may cause additional problems or indicate the existence of other dangerous conditions. In a population-based study^1^ on older adults without any heart failure signs or systolic dysfunction, the data collected by Holter monitor (median duration, 22.2 hours) show that 0.011% of all heart beats were PVCs, and 5.5% of participants had nonsustained VT. Over follow-up, baseline PVC percentage was significantly associated with an adjusted increased odds of decreased left ventricular ejection fraction (odds ratio, 1.13; 95% confidence interval, 1.05–1.21) and an increased adjusted risk of incident heart failure (hazard ratio, 1.06; 95% confidence interval, 1.02–1.09) and death (hazard ratio, 1.04; 95% confidence interval, 1.02–1.06). Idiopathic ventricular arrhythmia (IVA) is the common term used when referring to PVC and VT that occurred in the absence of structural heart disease. Cardiac catheter ablation has been proven as a reliable and effective therapy for IVAs and has been cited in the 2019 HRS, EHRA, APHRS, and LAHRS expert consensus statement^2^. The majority of IVA, outflow tract ventricular arrhythmias (OT-VAs) stem from either the right ventricular outflow tract (RVOT) or the left ventricular outflow tract (LVOT). Therefore, through analyzing the features of ECG, an accurate prediction of the OT-VA origins before the procedure can optimize the ablation result, reduce ablation duration, and avoid eventual operative complications. In fact, numerous studies^3–11^ have already revealed a strong relationship between the characteristics of ECG and the sites (RVOT or LVOT) where OT-VA stems from.

Moreover, ablation operators can use an analytical algorithm to predict OT-VA origins while optimizing the ablation procedure if the obtained characteristics of ECG can be used as the input to the given system or algorithm. Nevertheless, such a decision support system needs to be trained and validated by ECG data with accurate labels. To the best of our knowledge, such an ECG database is not available for scientific research yet. Under the auspices of Chapman University and Ningbo First Hospital of Zhejiang University, we created and shared a 12-lead ECG database that is intended to separate the origins of OT-VA from RVOT to LVOT. The data set is composed of 334 subjects who experienced OT-VA, and had the confirmative result of a successful catheter ablation procedure. Being the first database available for idiopathic ventricular arrhythmia studies, this resource can advance future research on OT-VA analysis.

## Methods

### Participants

The database consists of 334 patient ECGs including 104 males and 230 females. Among these patients, 257 (77%) have OT-VA originating from RVOT, and 77 (23%) are LVOT cases. Detailed baseline characteristics of all participants are presented in Table 1. The institutional review board of Ningbo First Hospital of Zhejiang University approved this study and allowed the data to be shared publicly after de-identification. The requirement for patient consent was waived. Patients were not informed of the study.

**Table 1:** Baseline characteristics of patients.

	All	RVOT	LVOT	P-Value
Patients, n (%)	334	257 (77)	77 (23)	<0.001
Age, Mean ± SD, year	46.1 ± 13.1	47.5 ± 13.4	46.2 ± 16.5	0.91
Male, n (%)	104 (32)	65 (27)	39 (49)	<0.001
Frequent PVC, n (%)	325 (98)	251 (99)	74 (97)	0.4357
Sustained VT, n (%)	9 (2)	6 (1)	3(3)	0.4357

### Classification of anatomic sites

**Fig. 1:**
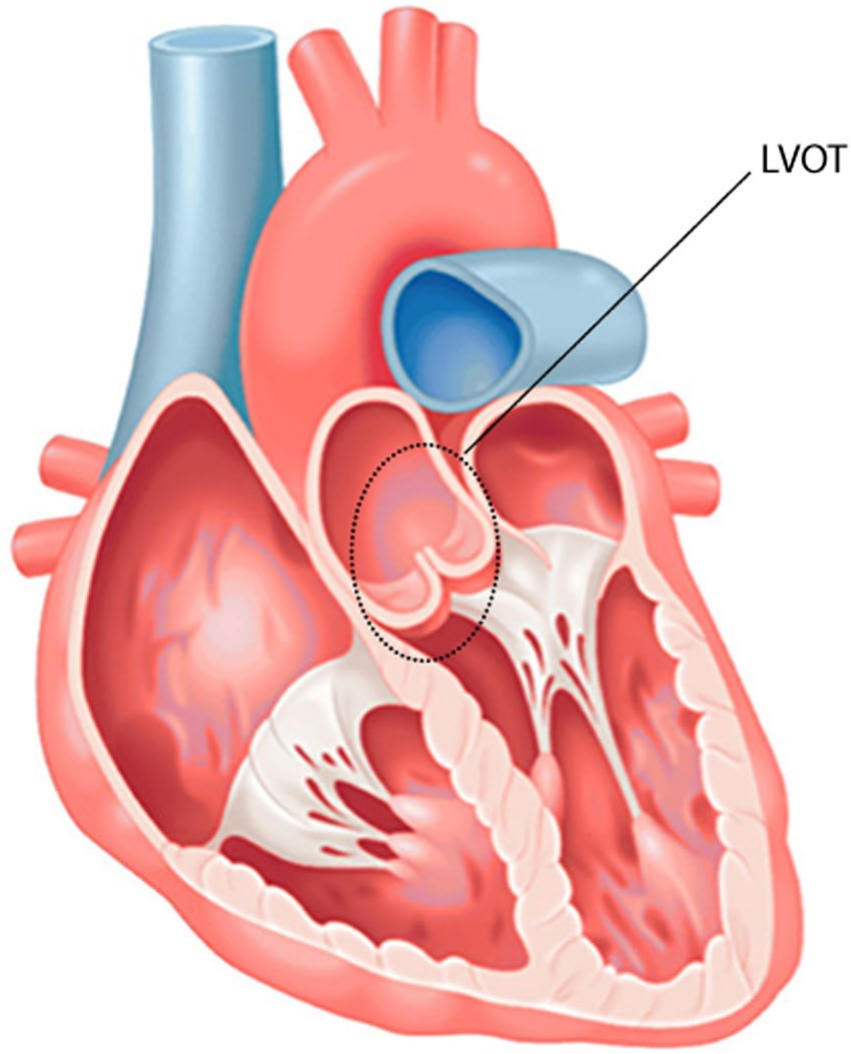
Anatomic location of LVOT. The left ventricular outflow tract (LVOT) which connects to the aorta is nearly indistinguishable from the rest of the ventricle.

**Fig. 2:**
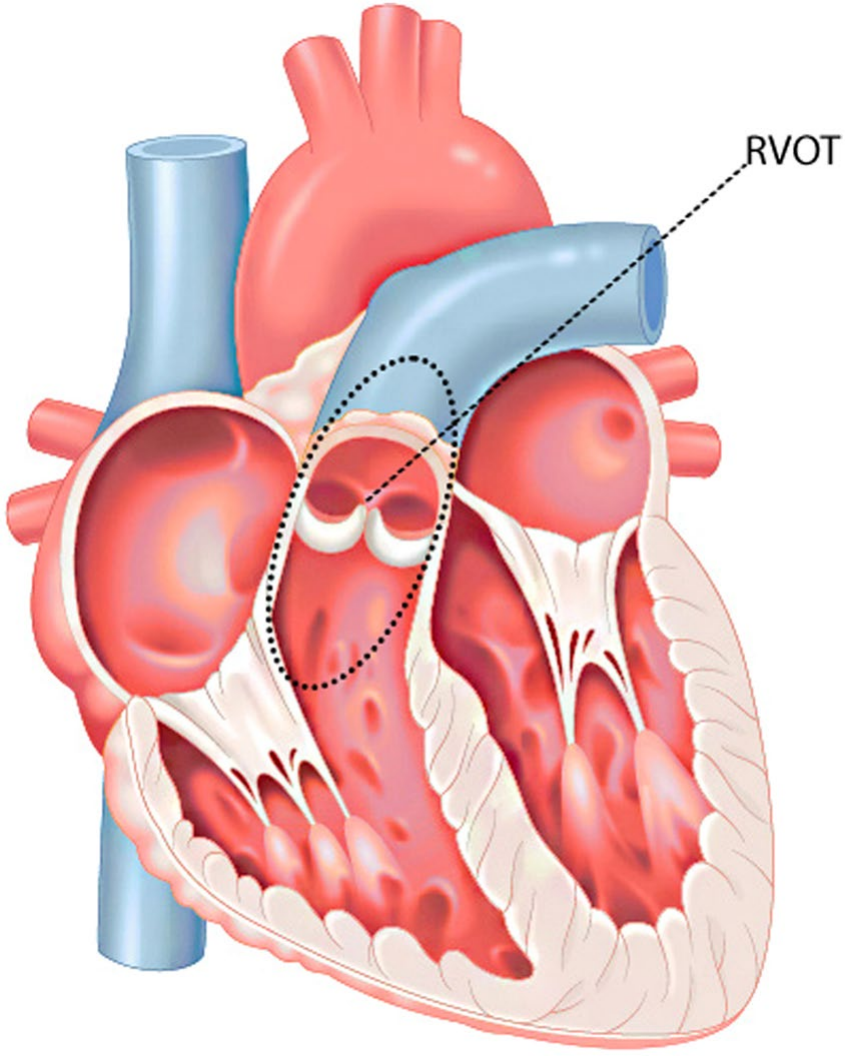
Anatomic location of RVOT. The right ventricular outflow tract (RVOT) is an infundibular extension of the ventricular cavity which connects to the pulmonary artery.

**Fig. 3:**
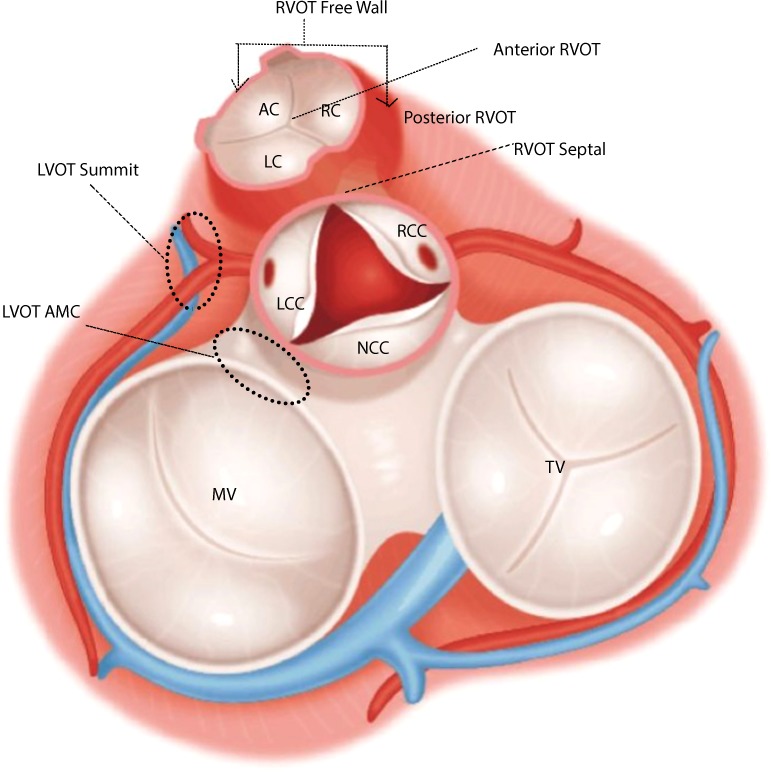
Sub anatomic sites in LVOT and RVOT. LCC = left coronary cusp; RCC = right coronary cusp; NCC = non-coronary cusp; AMC = aortomitral continuity; AC = anterior cusp; LC = left cusp; RC = right cusp; MV = mitral valve; TV = tricuspid valve.

In this work, the origins of OT-VA in the LVOT (shown in Fig. 1) are anatomically classified into 6 regions: left coronary cusp (LCC), right coronary cusp (RCC), non-coronary cusp (NCC), aortomitral continuity (AMC), summit, and LCC-RCC commissure respectively. The possible ablation sites in the RVOT (shown in Fig. 2) need to be identified by 3-dimensional directions: anterior and posterior, right and left, and superior and inferior. Accordingly, OT-VA origins in the RVOT are assigned into 7 regions: anterior cusp (AC), left cusp (LC), right cusp (RC), posterior septal, anterior septal, free wall, and other locations respectively. Figure 3 specifically illustrates all regions mentioned above, and the sample numbers associated with each anatomic site are shown in Table 2.

**Table 2:** Summary of anatomic sites.

Left/Right	Sub Locations	Number	Percentage
RVOT	LC	71	27.63%
RVOT	RVOTOther	45	17.51%
RVOT	Posterior Septal	32	12.45%
RVOT	AC	29	11.28%
RVOT	FreeWall	28	10.9%
RVOT	Anterior Septal	28	10.9%
RVOT	RC	24	9.34%
LVOT	LCC	39	50.05%
LVOT	AMC	18	23.38%
LVOT	RCC	7	9.09%
LVOT	LCC-RCC Ommisure	7	9.09%
LVOT	Summit	5	6.49%
LVOT	NA	1	1.29%

### Mapping and ablation procedure

**Fig. 4:**
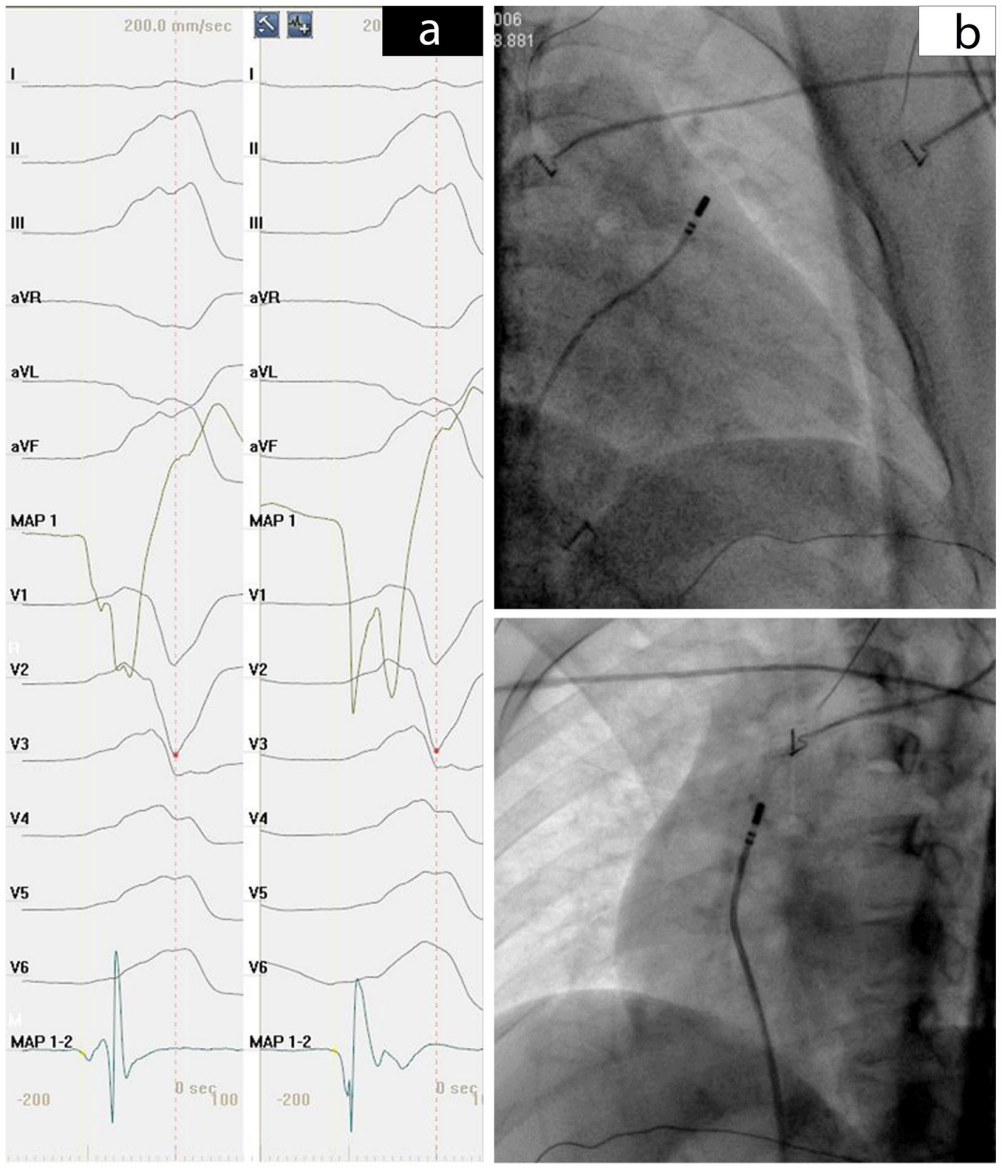
Acitivation map and fluoroscopy map for OT-VA originating from anterior septal in RVOT. (**a**) The earliest bipolar and unipolar activation time (−30 ms) was presented here. (**b**) Right anterior oblique and left anterior oblique fluoroscopic views showing an ablation catheter in the anterior interventricular vein (AIV) and another ablation catheter in the RVOT. Ablation in the RVOT (anterior septal) eliminated the PVC within 3 seconds.

**Fig. 5:**
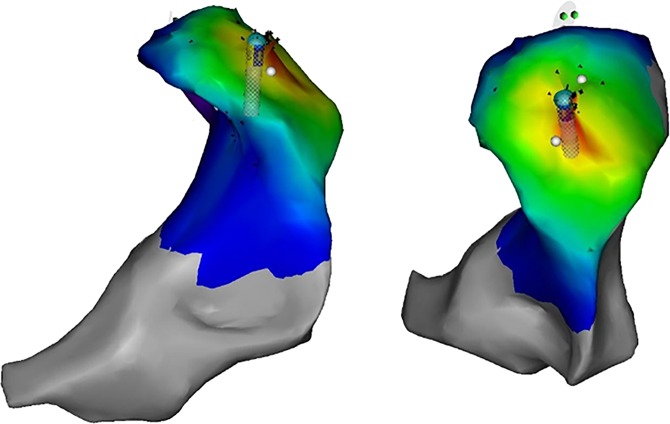
3D map for OT-VA originating from anterior septal in RVOT. The three-dimensional anatomic representation of the right ventricle, left ventricle, and venous system with the ablation catheter positioned at the AIV.

**Fig. 6:**
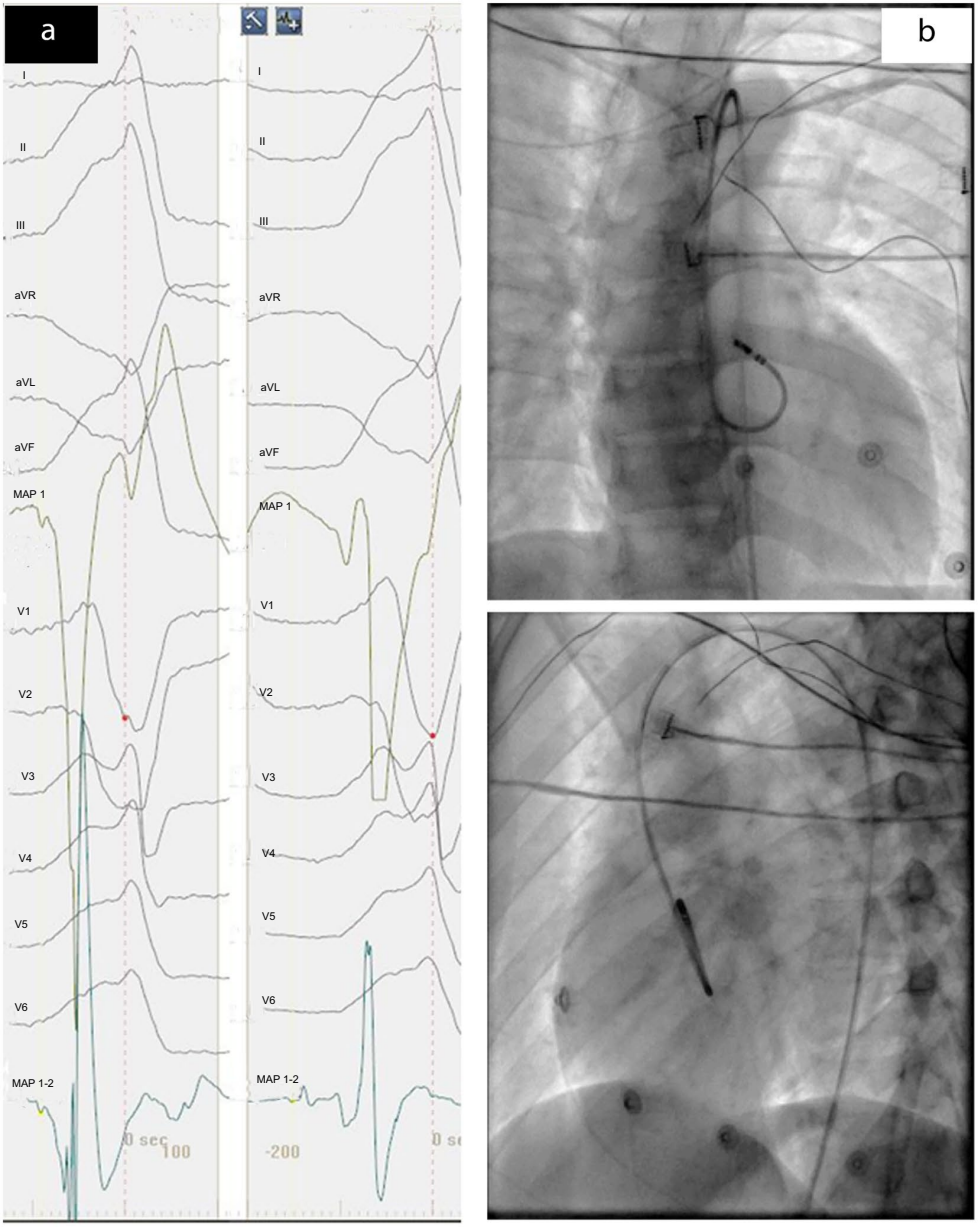
Acitivation map and fluoroscopic map for OT-VA originating from LCC-RCC commissure in LVOT. (**a**) The earliest bipolar and unipolar activation time ( − 30 ms) was presented here. (**b**) Right anterior oblique and left anterior oblique fluoroscopic views showing an ablation catheter in the anterior interventricular vein (AIV) and another ablation catheter in the LVOT. Ablation in the LVOT (LCC-RCC commissure) eliminated the PVC within 3 seconds.

**Fig. 7:**
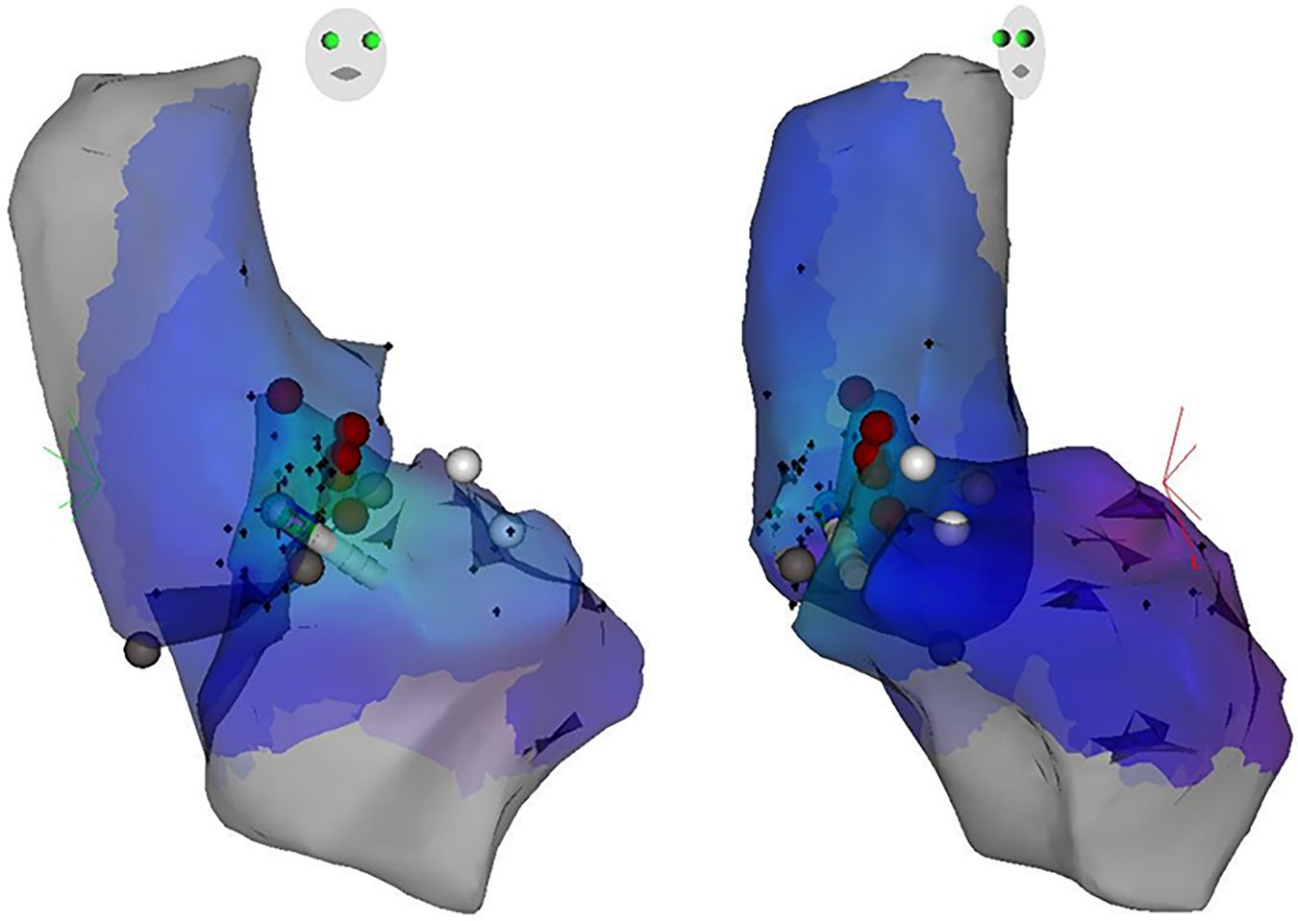
3D map for OT-VA originating from LCC-RCC commissure in LVOT. The three-dimensional anatomic representation of the right ventricle, left ventricle, and venous system with the ablation catheter positioned at the AIV.

Before the ablation procedure, antiarrhythmic drugs were ceased for at least 5 half-lives. The procedure was performed under the guidance of both fluoroscopy and 3-dimensional electroanatomic mapping system (CARTO, Biosense Webster, Diamond Bar, CA, USA). Moreover, activation mapping was performed in all patients during VT or PVCs. When VT or PVCs were infrequent, pace mapping was performed during sinus rhythm at a pacing cycle length of 500 milliseconds with the minimum stimulus amplitude required for consistent capture. Figures 4 and 5 respectively present the activation, fluoroscopy and 3-dimensional mapping example for the origin of anterior septal in RVOT. Furthermore, Figs. 6 and 7 depict a similar case of LCC-RCC commissure in LVOT. After the target site was located, the maximum radio frequency energy was delivered up to maximum power of 50 W and maximum eletrode-tissue interface temperature of 55 °C. If the VT or PVCs disappeared or their frequency diminished after the first 30 seconds of ablation, the energy was delivered continuously from 60 to 180 seconds. Ablation success was defined as the absence of spontaneous or induced OT-VAs at 30 minutes after the last energy delivery. The result has to be confirmed by continuous cardiac telemetry in the subsequent 24 hours of inpatient care.

### Data acquisition

During the whole ablation procedure, the 12-lead surface ECGs were collected by EP workmate system (EP-WorkMate^TM^ System, Abbott, Saint Paul, Minnesota, USA) at a sampling rate of 2000 Hz. In order to improve computation efficiency, a certain period of ECG that contains both normal heart beats and PVCs when OT-VAs occurred was truncated from the whole procedure and constituted this database. The diagnosis that indicated LVOT or RVOT were made according to the result of a successful ablation process. Consequently, each recorder can be a solid learning source to predict the OT-VA origins in RVOT or LVOT. Last but not least, only the OT-VAs that originated from a single source were included in this database, and the multi-source cases were excluded.

### Data denoising method

In this study, the noises presented in the proposed ECG database are power line interference, baseline wandering, and random noise. The Wavelets technique was used to remove the noises mentioned above. To get a full understanding of the technique and scheme that were adopted in this work, please refer to the Code Availability section.

#### Wavelet and multiresolution analysis

The wavelet transformation^12^ with multiresolution analysis (MRA) is a tool that splits up data into different frequency components, and then analyzes each component with a resolution associated with a customized time scale. Thus, wavelet transform can yield a better time-frequency localization result than windowed Fourier transform and naturally has an advantage in noise reduction applications. In this work, coif5 wavelet and SURE-based threshold were implemented. The denoising application based on wavelet desires to replace the decomposition coefficients under the estimated threshold with zeros which are supposed to represent noise components in the signal.

### Statistical analysis

For the continuous variable age, we calculated the mean and standard deviation. For all count variables, total sample size, number of males, number of subjects with frequent PVC and sustained VT, we calculated frequency counts and percentages. Detailed results are presented in Table 1. We compared the distributions of these background characteristics in the RVOT and LVOT groups and showed the p-values from the hypothesis testing procedures in the last column of this table. A one-sample test for proportion revealed that proportions of RVOT and LVOT are not equal among all cases (p-value < 0.001). This result is not surprising as the data were not obtained under a random sampling design. A two-sample t-test revealed that the average ages of subjects with RVOT and LVOT were not significantly different (p-value of 0.91). A two-sample test for proportions revealed that proportions of males (and females) in LVOT and RVOT groups were significantly different (p-value is < 0.001). A Fisher’s exact test revealed that proportions of subjects with frequent PVC (and sustained VT) in LVOT and RVOT groups were not significantly different (p-value of 0.44).

The percentages of sublocations within all RVOT and LVOT cases are shown in Table 2. The most frequent sublocations were LC and LCC for the RVOT and LVOT groups respectively. All analyses were done using R, version 3.5.3 (https://www.r-project.org).

## Data Records

**Fig. 8:**
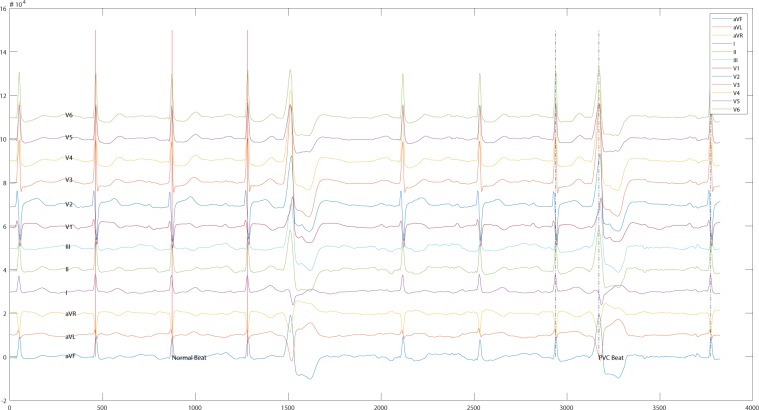
A segment of 12-lead ECG presents normal beat and PVC when OT-VA originating from LCC in LVOT.

**Fig. 9:**
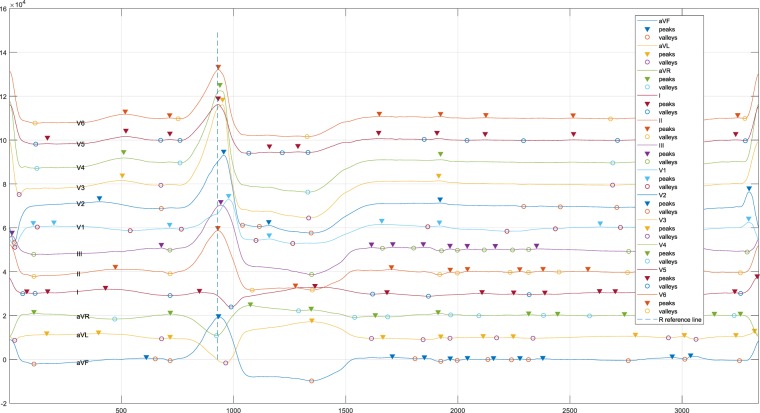
One PVC acquired during the catheter ablation procedure when OT-VA originating from LCC is in LVOT.

Data records presented in this work consist of three parts: raw ECG data, ECG data after noise reduction, and diagnostic file. These files are available online from figshare^13^. For each subject, the raw ECG data were saved into a single CSV file, and ECG data after noise reduction were done with the same name CSV file but in a different folder. Also, each CSV file mentioned above contains 12 columns with header names presenting the ECG leads. Figure 8 depicts a segment of a CSV file that contains normal heartbeats and PVCs when OT-VA occurred in LCC of LVOT. Sequentially, ECG representing single PVC (shown in Fig. 9) can be extracted for further analysis. These CSV files are named by unique IDs, and these IDs are also saved in the diagnostics file with an attribute name HospitalID. The diagnostics file contains all the diagnosis information (shown in Table 3) from each subject including HospitalID, Gender, Type, LeftRight, and Sublocation.

**Table 3:** Attributes in diagnosis file.

Attributes	Type	Description
HospitalID	String	ID is the primary key connected patient diagnoses with ECG data file
Type	String	Presents the condition patients experienced before abalation
Gender	String	The genotypical sex of the patient
LeftRight	String	Left means LVOT, and Rgith means RVOT
Sublocation	String	Sub locations shown in Table 2

## Technical Validation

### Ablation result validation

In the subsequent 24 hours of inpatient care after the ablation procedure, every patient took the ECG monitoring. After discharge, the patients underwent a follow-up two weeks after the ablation and then every month at the cardiology clinic. A 12-lead ECG test was conducted on each clinic visit, and 24 hour Holter monitoring also prescribed to each patient. The recorders were excluded if the recurrence of frequent PVCs or VT (happened above 5% of total test duration) in the first six-month follow-up was observed.

### Evaluation protocol for classification

Referring to the guidance from AHA, ACC, and HRS^14^, we proposed a five-step workflow for future study of origin sites classification.

#### Label selection

The available sites classification studies listed in^3,5–11^ were designed to distinguish LVOT and RVOT per patient. However, the sub locations under RVOT or LVOT are also important from a clinical prospective. Thus, the labels of this database are available to compare not only LVOT and LVOT, but also sub locations under them. Sites labels shown in Table 2 can composite different combinations according to different research purposes, but general pipeline and validation practice are suggested as follows for future work and comparison.

#### Processing

Following up with guidance from AHA, ACC and HRS^14^, we recommend a low-frequency filter to cut off 0.67Hz or below for linear digital filters with zero phase distortion, and a high-frequency filter with 150Hz cutoff frequency. Using the raw ECG signal is also an option for classification scheme.

#### Feature extraction and selection

The interpretable feature extraction method is recommended. Using feature selection method, one can report feature importance. Unsupervised feature selection, such as principal component analysis, is not suggested. We also recommend Neural Network models that use sequential transformations of the raw data as features that were ultimately fed into a multinomial logistic regression classifier (softmax unit).

#### Classification

We suggest 10-fold cross-validation for both super-parameter tuning and validation. Furthermore, the use of multiple numerical and sampling methods to improve classification performance, such as bootstrap and re-enforce training, is recommended. Finally, the classification result needs to report accuracy and performance on valuation dataset.

#### Evaluation

F_1_ score (1), Overall Accuracy (2), Confusion Matrix, Precision (3) (Positive Predictivity), and Recall (4) (Sensitivity) are recommended to report classification performance.

1 $${F}_{1}=2\ast \frac{Precision\ \ast \ Recall}{Precision+Recall}$$

2 $$Overall\ Accuracy=\frac{True\ Positive+True\ Negative}{Total\ Population}$$

3 $$Precision=\frac{True\ Positive}{True\ Positive+False\ Positive}$$

4 $$Recall=\frac{True\ Positive}{True\ Positive+False\ Negative}$$

## Usage Notes

We recommend a denoising implementation that is a Matlab program and can be found in Code Availability section. For ECG morphology characteristic measurement, BioSPPy (https://github.com/PIA-Group/BioSPPy/) is recommended to extract general ECG summary features such as QRS count, R wave location and others. As for machine learning packages, we suggest scikit-learn^15^ and TensorFlow (https://www.tensorflow.org/) for deep learning model building.

## Data Availability

The MATALB (https://www.mathworks.com/) program for ECG denoising is put under https://github.com/zheng120/PVCVTECGDenoising.

## References

[1] Benjamin J (2018). Heart Disease and Stroke Statistics-2018 Update: A Report From the American Heart Association. Circulation.

[2] Cronin EM (2019). 2019 HRS/EHRA/APHRS/LAHRS expert consensus statement on catheter ablation of ventricular arrhythmias: Executive summary. Heart Rhythm.

[3] Arya A (2011). Effect of limb lead electrodes location on ecg and localization of idiopathic outflow tract tachycardia: A prospective study. J. Cardiovasc. Electrophysiol..

[4] Betensky B (2011). The v2 transition ratio: A new electrocardiographic criterion for distinguishing left from right ventricular outflow tract tachycardia origin. JACC.

[5] Cheng D (2018). V3r/v7 index a novel electrocardiographic criterion for differentiating left from right ventricular outflow tract arrhythmias origins. Circ. Arrhythm Electrophysiol..

[6] Efimova E (2015). Differentiating the origin of outflow tract ventricular arrhythmia using a simple novel approach. Heart Rhythm.

[7] Ito S (2003). Development and validation of an ecg algorithm for identifying the optimal ablation site for idiopathic ventricular outflow tract tachycardia. J. Cardiovasc. Electrophysiol..

[8] Yoshida N (2011). Novel transitional zone index allows more accurate differentiation between idiopathic right ventricular outflow tract and aortic sinus cusp ventricular arrhythmias. Heart Rhythm.

[9] Yoshida N (2014). A novel electrocardiographic criterion for differentiating a left from right ventricular outflow tract tachycardia origin: The v2s/v3r index. J. Cardiovasc. Electrophysiol..

[10] Kamakura S (1998). Localization of optimal ablation site of idiopathic ventricular tachycardia from right and left ventricular outflow tract by body surface ecg. Circulation.

[11] Xie S (2018). Lead I r-wave amplitude to differentiate idiopathic ventricular arrhythmias with left bundle branch block right inferior axis originating from the left versus right ventricular outflow tract. J. Cardiovasc. Electrophysiol..

[12] Ingrid, D. Ten lectures on wavelets. *SIAM* (1992).

[13] Zheng J, Fu G, Anderson K, Chu H, Rakovski C (2019). figshare.

[14] Kligfield P (2007). Recommendations for the standardization and tnterpretation of the electrocardiogram. Circulation.

[15] Pedregosa F (2011). Scikit-learn: machine learning in Python. JMLR.

